# Clinical characteristics and prognosis of patients with COVID-19 on mechanical ventilation undergoing continuous renal replacement therapy

**DOI:** 10.1371/journal.pone.0297344

**Published:** 2024-04-03

**Authors:** Dae-Eun Choi, Duk Ki Kim, Sunghoon Park, Su Hwan Lee, Onyu Park, Taehwa Kim, Hye Ju Yeo, Jin Ho Jang, Woo Hyun Cho, Song I. Lee, On behalf of Korean Intensive Care Study Group

**Affiliations:** 1 Department of Nephrology, Chungnam National University Hospital, Chungnam National University School of Medicine, Daejeon, Republic of Korea; 2 Division of Allergy, Pulmonary, and Critical Care Medicine, Department of Internal Medicine, Chungnam National University Hospital, Chungnam National University School of Medicine, Daejeon, Republic of Korea; 3 Department of Pulmonary, Allergy, and Critical Care Medicine, Hallym University Sacred Heart Hospital, Anyang, Republic of Korea; 4 Division of Pulmonology, Department of Internal Medicine, Severance Hospital, Yonsei University College of Medicine, Seoul, Republic of Korea; 5 College of Nursing, Research Institute of Nursing Science, Pusan National University, Yangsan, Republic of Korea; 6 Division of Allergy, Pulmonary, and Critical Care Medicine, Department of Internal Medicine, Pusan National University Yangsan Hospital, Pusan National University School of Medicine, Busan, Republic of Korea; Pikeville Medical Center, UNITED STATES

## Abstract

**Background:**

The coronavirus disease (COVID-19) pandemic has significantly strained global healthcare, particularly in the management of patients requiring mechanical ventilation (MV) and continuous renal replacement therapy (CRRT). This study investigated the characteristics and prognoses of these patients.

**Methods:**

This multicenter retrospective cohort study gathered data from patients with COVID-19 across 26 medical centers. Logistic analysis was used to identify the factors associated with CRRT implementation.

**Results:**

Of the 640 patients with COVID-19 who required MV, 123 (19.2%) underwent CRRT. Compared to the non-CRRT group, the CRRT group was older and exhibited higher sequential organ failure assessment scores. The incidence of hypertension, diabetes, cardiovascular disease, chronic neurological disease, and chronic kidney disease was also higher in the CRRT group. Moreover, the CRRT group had higher intensive care unit (ICU) (75.6% vs. 26.9%, p < 0.001) and in-hospital (79.7% vs. 29.6%, p < 0.001) mortality rates. CRRT implementation was identified as an independent risk factor for both ICU mortality (hazard ratio [HR]:1.833, 95% confidence interval [CI]:1.342–2.505, p < 0.001) and in-hospital mortality (HR: 2.228, 95% CI: 1.648–3.014, p < 0.001). Refractory respiratory failure (n = 99, 19.1%) was the most common cause of death in the non-CRRT death group, and shock with multi-organ failure (n = 50, 40.7%) was the most common cause of death in the CRRT death group. Shock with multi-organ failure and cardiac death were significantly more common in the CRRT death group, compared to non-CRRT death group.

**Conclusion:**

This study indicates that CRRT is associated with higher ICU and in-hospital mortality rates in patients with COVID-19 who require MV. Notably, the primary cause of death in the CRRT group was shock with multi-organ failure, emphasizing the severe clinical course for these patients, while refractory respiratory failure was most common in non-CRRT patients.

## Introduction

The coronavirus disease (COVID-19) pandemic, caused by severe acute respiratory syndrome coronavirus 2 (SARS-CoV-2), was declared a global pandemic by the World Health Organization on March 11, 2020 [1]. Approximately 20% of patients with COVID-19 experienced severe disease that required hospitalization [2–5]. The percentage of hospitalized patients admitted to the intensive care unit (ICU) varied depending on the variant of COVID-19 and country; in some cases, it was as high as 40% [3, 4, 6–9]. In these cases, patients admitted to the ICU often required invasive mechanical ventilation (MV) because of acute respiratory distress syndrome (ARDS) or dialysis for acute kidney injury (AKI).

Kidney injury in COVID-19 infection is mechanistically influenced by various complex factors [10–13]. These include direct viral injury, as SARS-CoV-2 infects kidney cells via ACE2 receptors [10–12]. In addition, indirectly, the following mechanisms are involved; respiratory infections and inflammation affecting the renal vasculature [11–13]; cytokine storms contributing to renal dysfunction [11–13]; increased blood clot formation impacting kidney blood vessels [11–13]; and the presence of pre-existing conditions that exacerbate kidney damage when combined with COVID-19 [13]. The incidence of kidney injury in patients with COVID-19 ranges from 8.9%–34% [14–16]. Severe COVID-19 is associated with a high likelihood of kidney injury and often necessitates continuous renal replacement therapy (CRRT) [17]. Moreover, although there are small size studies, patients requiring CRRT were predicted to have increased mortality rates than those not-requiring CRRT in severe covid patients with mechanical ventilation [18, 19]. However, detailed information pertaining to the characteristics and prognosis of these patients remains limited. In this study, we aimed to evaluate the characteristics and prognosis of patients with COVID-19 who underwent MV and CRRT.

## Materials and methods

### Ethics statement

This study was approved by the Institutional Review Board (no. 2021-12-044). Because of the retrospective nature of the study, the requirement for informed consent was waived.

### Study design

This multicenter retrospective cohort study investigated the medical records of adult patients aged ≥19 years who were diagnosed with COVID-19 and received treatment beyond a high-flow nasal cannula (HFNC) in 26 medical institutions (S1 Table) to treat severely to critically ill patients with COVID-19 from January 2020 to August 2021. Data were retrieved from electronic medical records and were anonymized subsequent to collection. Analysis was conducted upon access to the anonymized data on February 28, 2023. This investigation adhered to the principles of the Declaration of Helsinki and was conducted in line with the STROBE reporting guidelines.

We collected medical data on patient age, underlying comorbidities, clinical frailty scale scores, COVID-19-related symptoms and signs, administered therapeutic agents, laboratory findings, utilized oxygen devices, details regarding MV, implementation of CRRT, patient outcomes, ICU admission status, and duration of stay in the ICU and hospital. Patient severity was assessed based on the sequential organ failure assessment (SOFA) score at the time of the initial application of MV [20].

### Definition and indication of the device

COVID-19 was diagnosed as an acute SARS-CoV-2 infection by performing a nucleic acid amplification test or an antigen test on samples collected from the upper respiratory tract. HFNC was used when oxygen saturation in patients with severe COVID-19 could not be maintained above 90% with conventional oxygen therapy. Invasive MV was administered to critically ill patients with COVID-19 in cases in which oxygen saturation could not be maintained above 90%, despite using HFNC or when vital signs were unstable [21, 22]. The decision to initiate CRRT and the selection between continuous venovenous hemofiltration (CVVH) and continuous venovenous hemodiafiltration (CVVHDF) were made by the attending intensivist and nephrologist. Typically, CRRT was initiated based on factors [23]. CRRT initiation was deferred until deemed necessary, and the specific modality (CVVH or CVVHDF) was chosen based on the patient’s clinical condition and therapeutic goals. In our study, the non-CRRT group was defined as patients who either did not develop acute kidney injury, or, even when acute kidney injury was present, did not undergo CRRT.

### Statistical analysis

All categorical variables were presented as percentages, whereas continuous variables were expressed as median (interquartile range [IQR]: 25^th^–75^th^ percentile) and mean ± standard deviation. Student’s t-test was used to analyze continuous data, whereas Pearson’s chi-square test or Fisher’s exact test was used to analyze categorical data. Logistic regression analysis was conducted to assess the factors associated with CRRT, and Cox regression analysis was performed to evaluate the associated factors for mortality. In the univariate analysis, factors with a p < 0.05 were identified and included in the multivariate analysis. The risk factors for CRRT were represented as odds ratios (OR) and 95% confidence intervals (CI), and the factors associated with in-hospital mortality were presented as hazard ratios (HR) and 95% CI. Statistical significance was defined as p < 0.05. In addition, the survival rate of patients based on CRRT implementation was analyzed using Kaplan-Meier survival analysis. The Statistical Package for the Social Sciences software (version 22.0; IBM Corporation, Somers, NY, USA) was used for all statistical analyses.

## Results

### Enrolled patient characteristics

**Fig 1:**
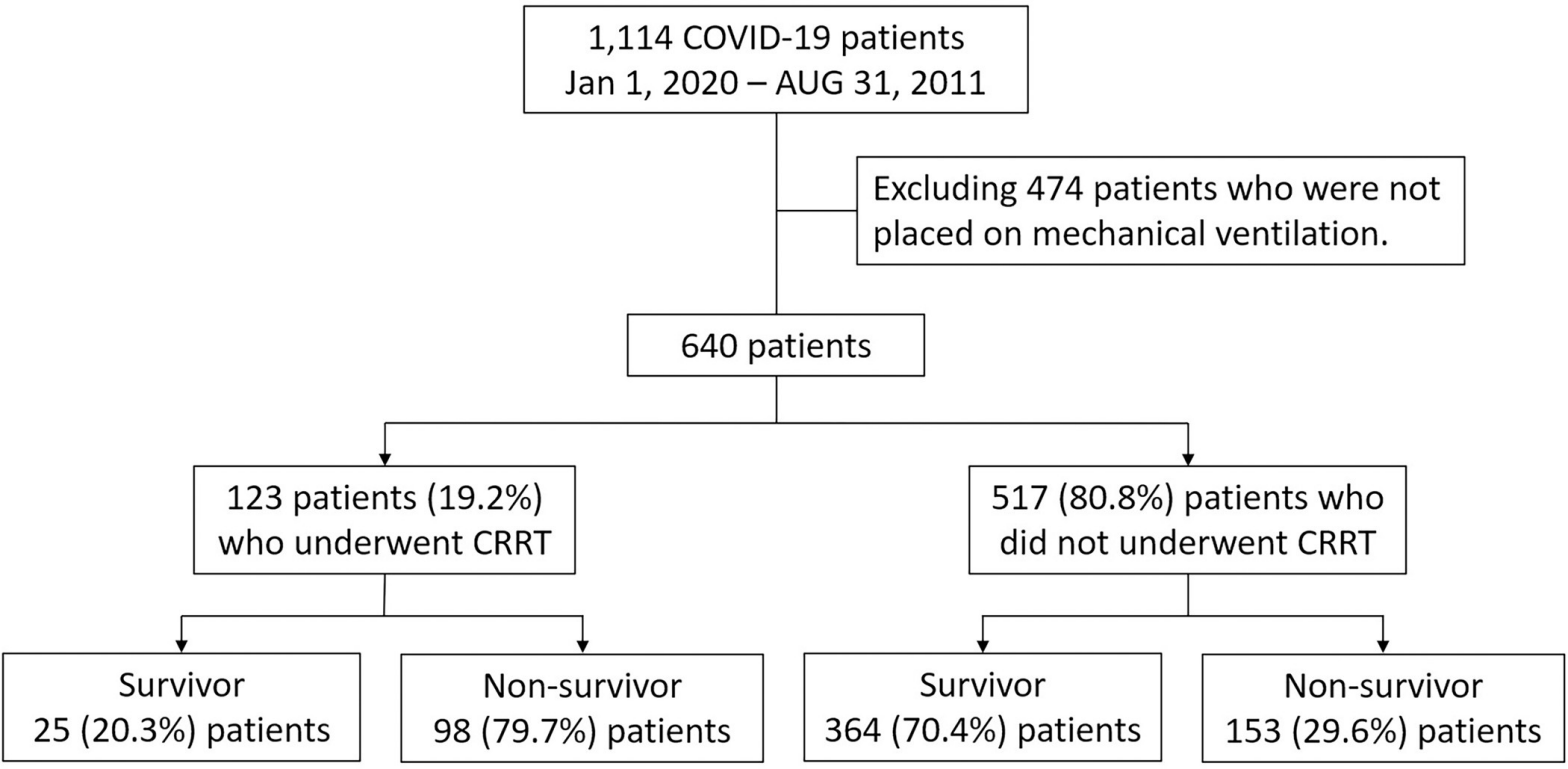
Flow diagram representing patient inclusion.

During the study period, 1,114 patients were screened. Of these, 640 were included after excluding 474 patients who were not on MV (Fig 1). Of these patients, 123 (19.2%) underwent CRRT, whereas 517 (80.8%) did not.

In the CRRT group, patients were older (71.4 ± 13.0 vs. 68.5 ± 12.7, p = 0.022) and had higher SOFA scores (9.2 ± 3.4 vs. 7.3 ± 3.1, p < 0.001) than the non-CRRT group. Among underlying comorbidities, hypertension (68.3% vs. 54.4%, p = 0.005), diabetes (44.7% vs. 33.7%, p = 0.021), cardiovascular disease (17.9% vs. 10.4%, p = 0.022), chronic neurological disease (19.5% vs. 12.4%, p = 0.039), and chronic kidney disease (CKD; 25.2% vs. 4.1%, p < 0.001) were more frequently associated with the CRRT group than the non-CRRT group (Table 1).

**Table 1:** Baseline characteristics of enrolled patients.

Variables	All patients (n = 640)	Non-CRRT (n = 517)	CRRT (n = 123)	p-value
Age	69.1 ± 12.8	68.5 ± 12.7	71.4 ± 13.0	0.022
Male (%)	387 (60.5)	305 (59.0)	82 (66.7)	0.118
Smoking (%)	139 (21.7)	111 (21.5)	28 (22.8)	0.075
Body mass index	25.0 ± 4.1	24.8 ± 4.0	25.6 ± 4.7	0.075
Clinical frailty scale	3.0 (2.0–4.0)	3.0 (2.0–4.0)	3.0 (2.0–4.0)	0.775
SOFA score	7.6 ± 3.3	7.3 ± 3.1	9.2 ± 3.4	<0.001
Vaccination hx.	29 (4.5)	27 (5.2)	2 (1.6)	0.127
Comorbidity (%)				
Hypertension	365 (57.0)	281 (54.4)	84 (68.3)	0.005
Diabetes	229 (35.8)	174 (33.7)	55 (44.7)	0.021
Cardiovascular disease	76 (11.9)	54 (10.4)	22 (17.9)	0.022
Chronic lung disease	52 (8.1)	46 (8.9)	6 (4.9)	0.143
Chronic neurological disease	88 (13.8)	64 (12.4)	24 (19.5)	0.039
Chronic kidney disease	52 (8.1)	21 (4.1)	31 (25.2)	<0.001
Chronic liver disease	20 (3.1)	18 (3.5)	2 (1.6)	0.288
Hematologic malignancy	11 (1.7)	11 (2.1)	0 (0)	0.103
Solid organ tumor	48 (7.5)	38 (7.4)	10 (8.1)	0.768

Data are presented as mean ± standard deviation or number (%), unless otherwise indicated.

SOFA: Sequential Organ Failure Assessment

The vital signs did not differ significantly between the two groups. However, in terms of laboratory findings, the CRRT group showed higher white blood cells (WBC) count, blood urea nitrogen (BUN), and creatinine levels and lower albumin levels. Additionally, arterial blood gas analysis revealed lower pH in the CRRT group than in the non-CRRT group (S2 Table).

### Factors related to patients needing CRRT

Multivariate logistic regression analysis showed that the factors associated with patients requiring CRRT included a high body mass index (BMI; OR: 1.075, 95% CI: 1.020–1.133, p = 0.007), high SOFA score (OR 1.088, 95% CI: 1.012–1.168, p = 0.022, presence of CKD (OR: 2.770, 95% CI: 1.343–5.713, p = 0.006), and elevated WBC count (OR: 1.036, 95% CI: 1.006–1.067, p = 0.019) and BUN level (OR: 1.037, 95% CI: 1.023–1.051, p < 0.001) (Table 2).

**Table 2:** Univariate and multivariate risk factors associated with CRRT (logistic regression analysis).

	Univariate analysis			Multivariate analysis		
	OR	95% CI	P-value	OR	95% CI	P-value
Age	1.019	1.003–1.036	0.023	1.014	0.995–1.034	0.148
Male	0.719	0.476–1.088	0.119			
Body mass index	1.049	1.000–1.099	0.048	1.075	1.020–1.133	0.007
Clinical frailty scale	1.018	0.903–1.147	0.774			
SOFA score	1.194	1.122–1.271	<0.001	1.088	1.012–1.168	0.022
Comorbidity						
Hypertension	1.809	1.192–2.746	0.005	1.049	0.631–1.743	0.855
Diabetes	1.594	1.069–2.377	0.022	1.026	0.629–1.675	0.917
Cardiovascular disease	1.868	1.088–3.206	0.023	1.124	0.578–2.183	0.731
Chronic neurologic disease	1.716	1.023–2.878	0.041	1.204	0.641–2.261	0.564
Chronic kidney disease	7.959	4.381–14.457	<0.001	2.770	1.343–5.713	0.006
Laboratory findings						
White blood cell, 10^3^/uL	1.055	1.023–1.089	0.001	1.036	1.006–1.067	0.019
Albumin, g/dL	0.606	0.418–0.878	0.008	0.752	0.478–1.184	0.218
BUN, mg/dL	1.051	1.039–1.064	<0.001	1.037	1.023–1.051	<0.001
Creatinine, mg/dL	1.819	1.509–2.192	<0.001	1.130	0.947–1.349	0.176
C-reactive protein, mg/dL	1.000	0.997–1.003	0.815			
Lactate, mmol/L	1.034	0.976–1.096	0.259			

OR: Odds ratio, CI: Confidence interval, SOFA: Sequential Organ Failure Assessment, BUN: blood urea nitrogen

### Patient treatment and clinical outcomes

**Fig 2:**
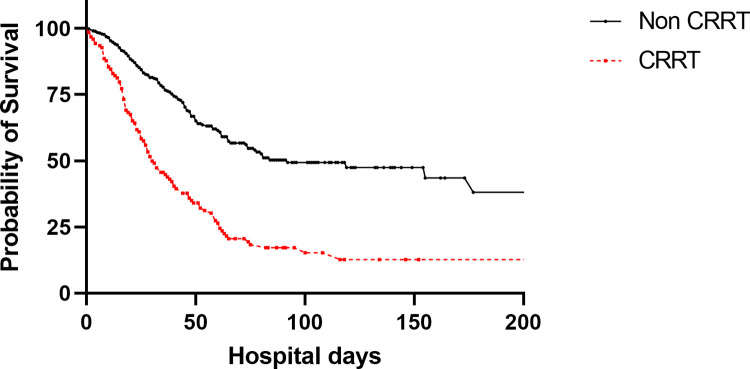
Kaplan-Meier curves of patient groups according to whether or not they received continuous renal replacement therapy (CRRT).

The therapeutic agents administered did not differ significantly between the two groups. However, the CRRT group had a higher frequency of extracorporeal membrane oxygenation (43.1% vs. 14.3%, p < 0.001) and tracheostomy (48.0% vs. 30.2%, p < 0.001) than the non-CRRT group. Additionally, the CRRT group exhibited higher rates of ICU mortality (75.6% vs. 26.9%, p < 0.001), in-hospital mortality (79.7% vs. 29.6%, p < 0.001), and a greater prevalence of issues related to the limitation of life-sustaining treatment (LST; 47.2% vs. 24.0%, p < 0.001) than the non-CRRT group (Table 3). When analyzing survival using Kaplan-Meier curves based on the implementation of CRRT, the CRRT group demonstrated a lower survival rate than the non-CRRT group (log-rank test, p < 0.001; Fig 2).

**Table 3:** Treatment and clinical outcomes.

Variables	All patients (n = 640)	Non-CRRT (n = 517)	CRRT (n = 123)	p-value
Treatment (%)				
Remdesivir	426 (66.6)	349 (67.5)	77 (62.6)	0.300
Steroid	614 (95.9)	493 (95.4)	121 (98.4)	0.128
Tocilizumab	56 (8.8)	46 (8.9)	10 (8.1)	0.787
Convalescent plasma	23 (3.6)	19 (3.7)	4 (3.3)	0.821
O2 device				
HFNC	434 (67.8)	360 (69.6)	74 (60.2)	0.043
ECMO	127 (19.8)	74 (14.3)	53 (43.1)	<0.001
Non-invasive ventilation	53 (8.3)	45 (8.7)	8 (6.5)	0.426
Tracheostomy	215 (33.6)	156 (30.2)	59 (48.0)	<0.001
Outcomes				
ICU mortality	232 (36.3)	139 (26.9)	93 (75.6)	<0.001
ICU LOS	22.0 (13.0–40.0)	21.0 (12.5–39.5)	26.0 (14.0–45.0)	0.263
In-hospital mortality	251 (39.2)	153 (29.6)	98 (79.7)	<0.001
Hospital LOS	30.0 (19.0–55.0)	30.0 (20.0–53.0)	29.0 (17.0–59.0)	0.490
Limitation of life sustaining treatment	182 (28.4)	124 (24.0)	58 (47.2)	<0.001

Data are presented as mean ± standard deviation or number (%), unless otherwise indicated.

HFNC: High flow nasal cannula, ECMO: Extracorporeal membrane oxygenation, ICU: Intensive care unit, LOS: length of stay

### Factors related to ICU and in-hospital mortality

Multivariate Cox regression analysis revealed factors associated with ICU mortality, including elevated creatinine (HR: 1.119, 95% CI: 1.034–1.211, p = 0.005) and lactate (HR: 1.049, 95% CI: 1.013–1.087, p = 0.007) levels, along with the implementation of CRRT (HR: 1.833, 95% CI: 1.342–2.505, p < 0.001) and the presence of LST issues (HR: 4.358, 95% CI: 3.200–5.935, p < 0.001) (Table 4).

**Table 4:** Univariate and multivariate risk factors associated with ICU mortality. (Cox regression analysis).

	Univariate analysis			Multivariate analysis		
	HR	95% CI	P-value	HR	95% CI	P-value
Age	1.025	1.013–1.037	<0.001	1.006	1.013–1.087	0.387
Male	1.023	0.782–1.339	0.867			
Body mass index	1.014	0.982–1.047	0.411			
Clinical frailty scale	1.099	1.019–1.186	0.015	1.016	0.916–1.127	0.767
SOFA score	1.059	1.017–1.103	0.005	1.005	0.958–1.053	0.846
Comorbidity						
Hypertension	1.090	0.838–1.417	0.520			
Diabetes	1.046	0.803–1.362	0.741			
Chronic lung disease	1.253	0.831–1.887	0.281			
Chronic kidney disease	1.518	1.027–2.246	0.036	1.020	0.586–1.775	0.944
Solid tumor	1.476	0.959–2.272	0.077			
Laboratory findings						
White blood cell, 10^3^/uL	1.001	0.989–1.013	0.851			
Hemoglobin, g/dL	0.941	0.882–1.004	0.067			
Platelet, 10^3^/uL	0.998	0.996–1.000	0.031	1.000	0.998–1.002	0.922
Albumin, g/dL	0.992	0.776–1.269	0.952			
Bilirubin, mg/dL	1.209	0.978–1.495	0.079			
Creatinine, mg/dL	1.137	1.073–1.204	<0.001	1.119	1.034–1.211	0.005
C-reactive protein, mg/dL	1.001	0.999–1.002	0.297			
P/F ratio, mmHg	0.999	0.998–1.001	0.291			
Lactate, mmol/L	1.080	1.046–1.115	<0.001	1.049	1.013–1.087	0.007
Treatment						
Steroid	0.903	0.445–1.830	0.776			
Tocilizumab	1.046	0.653–1.675	0.851			
CRRT	2.434	1.871–3.168	<0.001	1.833	1.342–2.505	<0.001
LST issue	4.932	3.747–6.490	<0.001	4.358	3.200–5.935	<0.001

HR: Hazard ratio, CI: Confidence interval, SOFA: Sequential Organ Failure Assessment, P/F ratio: arterial partial pressure of oxygen/inspired oxygen concentration ratio, CRRT: continuous renal replacement therapy, LST: life sustaining treatment

The factors associated with in-hospital mortality after multivariate analysis included elevated bilirubin (HR: 1.391, 95% CI: 1.124–1.723, p = 0.002) and creatinine (HR: 1.118, 95% CI: 1.037–1.207, p = 0.004) levels, implementation of CRRT (HR: 2.228, 95% CI: 1.648–3.014, p < 0.001), and the presence of LST issues (HR: 6.084, 95% CI: 4.509–8.210, p < 0.001) (S3 Table).

### Differences in clinical characteristics between CRRT and non-CRRT death

In the CRRT death group, patients had higher SOFA scores (9.0 ± 3.5 vs. 7.8 ± 3.3, p = 0.008) than the non-CRRT death group. Among underlying comorbidities, hypertension (67.3% vs. 54.2%, p = 0.039), and CKD (22.4% vs. 6.5%, p < 0.001) were more frequently associated with the CRRT death group than the non-CRRT death group. However, Chronic lung disease (5.1% vs. 14.4%, p = 0.021), and Hematologic malignancy (0% vs. 3.9%, p = 0.047) were more frequently associated with the non-CRRT death group than the CRRT death group (Table 5). In addition, the CRRT death group showed higher creatinine levels and lower arterial pH, compared to non-CRRT death group (S4 Table).

**Table 5:** Baseline characteristics of enrolled patients–non-survived patients.

Variables	All patients (n = 251)	Non-CRRT (n = 153)	CRRT (n = 98)	p-value
Age	73.3 ± 11.5	73.7 ± 11.0	72.6 ± 12.4	0.450
Male (%)	157 (62.5)	92 (60.1)	65 (66.3)	0.322
Smoking (%)	53 (21.1)	31 (20.3)	22 (22.4)	0.679
Body mass index	24.9 ± 4.0	24.7 ± 3.8	25.3 ± 4.4	0.194
Clinical frailty scale	3.0 (2.0–4.0)	3.0 (2.0–4.0)	3.0 (2.0–4.0)	0.077
SOFA score	8.3 ± 3.4	7.8 ± 3.3	9.0 ± 3.5	0.008
Comorbidity (%)				
Hypertension	149 (59.4)	83 (54.2)	66 (67.3)	0.039
Diabetes	93 (37.1)	50 (32.7)	43 (43.9)	0.073
Cardiovascular disease	38 (15.1)	21 (13.7)	17 (17.3)	0.435
Chronic lung disease	27 (10.8)	22 (14.4)	5 (5.1)	0.021
Chronic neurological disease	43 (17.1)	23 (15.0)	20 (20.4)	0.270
Chronic kidney disease	32 (12.7)	10 (6.5)	22 (22.4)	<0.001
Chronic liver disease	9 (3.6)	7 (4.6)	2 (2.0)	0.292
Hematologic malignancy	6 (2.4)	6 (3.9)	0 (0)	0.047
Solid organ tumor	27 (10.8)	17 (11.1)	10 (10.2)	0.821

Data are presented as mean ± standard deviation or number (%), unless otherwise indicated.

SOFA: Sequential Organ Failure Assessment

In the causes of death, refractory respiratory failure (n = 99, 19.1%) was the most common cause of death in the non-CRRT death group, and shock with multi-organ failure(n = 50, 40.7%) was the most common cause of death in the CRRT death group. Shock with multi-organ failure and cardiac death were significantly more common in the CRRT death group, compared to non-CRRT death group. On the other hand, there was no significant difference in the case of death due to refractory respiratory failure between CRRT death group and non-CRRT death group (Table 6).

**Table 6:** Cause of death by group.

Variables	All patients (n = 640)	Non-CRRT (n = 517)	CRRT (n = 123)	p-value
In-hospital mortality	251 (39.2)	153 (29.6)	98 (79.7)	<0.001
Refractory respiratory failure	131 (20.5)	99 (19.1)	32 (26.0)	0.090
Shock with multi-organ failure	91 (14.2)	41 (7.9)	50 (40.7)	<0.001
Cardiac death	8 (1.3)	3 (0.6)	5 (4.1)	0.002
Neurologic death	2 (0.3)	2 (0.4)	0 (0)	0.490
Others†	19 (3.0)	8 (1.5)	11 (8.9)	<0.001

†Death of undetermined etiology

## Discussion

In this study, 19.2% of the critical patients with COVID-19 on MV required CRRT. The CRRT group had higher ICU and in-hospital mortality rates, indicating a significant association with patient survival.

The CRRT group comprised older patients with higher SOFA scores and a higher prevalence of underlying comorbidities, including hypertension, diabetes, cardiovascular disease, chronic neurological disease, and CKD. Fominskiy et al. [18] showed that the renal replacement therapy (RRT) group had a higher proportion of older individuals and a greater prevalence of moderate/severe CKD. Lin et al. [17] reported a higher incidence of kidney injury in patients aged 60 years or older than the younger age group (60.7% vs. 39.3%). Similarly, Sullivan et al. [14] showed that patients who received kidney replacement therapy had a higher prevalence of diabetes, CKD, and obesity. Further, Ng et al. [24] observed that patients with kidney injury had a higher prevalence of comorbidities such as diabetes, hypertension, coronary artery disease, and heart failure. Consequently, patients who undergo CRRT are often older and have a higher prevalence of underlying comorbidities. In addition, elevated WBC, serum Creatinine, and BUN levels and lower arterial pH were observed in these patients. These finding conclusively suggested that AKI and accompanying acidosis were more common in the CRRT group. Also, elevated WBC has been known as the most efficient indicators of critical disease with COVID 19 [25]. Moreover, a previous study reported a positive correlation between COVID-19 severity and the need for RRT [17].

In this study, the factors associated with CRRT utilization included high BMI, CKD, and elevated WBC counts and BUN levels in COVID 19-patients with MV. It has been known that higher BMI is associated with higher incidence of AKI in ARDS patients [26]. Moreover, studies have identified additional factors linked to kidney injury that necessitate RRT, including CKD [14], history of transplantation [27], and high creatinine levels [28]. Higher stages of AKI are often associated with higher risk of hypercatabolism. BUN may be evidence of hypercatabolism in severe AKI [29]. In multivariate Cox regression analysis, CRRT utilization was significantly associated with poor prognosis in patients with COVID-19 compared with the non-CRRT group. Additionally, elevated creatinine and lactate levels and the presence of issues related to limitation of LST were associated with ICU mortality in patients with COVID-19 on MV. Previously, the mortality in patients with COVID-19 who underwent RRT was found to be similar [18] or even higher than those without RRT [14, 30–32]. In addition, it has been known that high level of lactate is a predictor of mortality in patients undergoing CRRT for acute kidney injury [33].

In the analysis of deceased patients, CRRT death group had a high SOFA score, underlying CKD, high WBC and serum Cr, and low arterial pH compared to non-CRRT death group. On the other hand, non-CRRT death group had significantly more underlying chronic lung disease compared to CRRT death group. Main causes of death are refractory respiratory failure in the non-CRRT patients, and shock with multi-organ failure in the CRRT patients, respectively. Despite more chronic lung disease in non-CRRT death group, patients who died from refractory respiratory failure were not significantly different from CRRT death group. It can be inferred that reflactory respiratory failure occurs more in patients who require CRRT even in the absence of underlying chronic lung disease. In previous studies, occurrence of AKI increases the need for MV due to respiratory failure [19] and is also associated with severity of COVID19 [32]. Also, similar to our results, people who do CRRT are more likely to experience shock with multi organ failure [34] and cardiac death [35]. In our study, patients with COVID-19 treated with both CRRT and MV had a higher likelihood of underlying CKD and a higher severity score, suggesting a higher probability of poor prognosis. Furthermore, studies have indicated a higher likelihood of requiring dialysis after discharge among patients who underwent RRT and had CKD [24], highlighting the possibility of continuous treatment and follow-up care, even after hospital discharge.

In the group of non-survived patients, there were notable differences in baseline characteristics and causes of death depending on the implementation of CRRT. One of the notable observations in our study is the higher SOFA scores among patients in the CRRT death group compared to the non-CRRT death group. This implies a more severe organ dysfunction in the former, possibly contributing to the decision to initiate CRRT [36, 37]. These findings highlight the importance of early risk stratification and consideration of CRRT in patients with higher SOFA scores to optimize their management [38]. Furthermore, the presence of underlying comorbidities played a significant role in determining patient outcomes. Hypertension and Chronic Kidney Disease (CKD) were more prevalent in the CRRT death group. These comorbidities are known to be risk factors for COVID-19 outcomes [39, 40], and their association with the CRRT death group underscores the need for vigilant monitoring and early intervention in such patients.

The primary causes of death in COVID-19, as widely recognized, are respiratory failure [41, 42] and multiple organ failure [43]. In our study, refractory respiratory failure was the predominant cause of death in the non-CRRT group, which aligns with the typical progression of severe respiratory diseases. In the CRRT group, however, the leading cause of death was shock with multi-organ failure, indicating the complexity and severity of cases requiring CRRT [44]. Notably, cardiac deaths were also higher in the CRRT group, suggesting the need for vigilant cardiac monitoring in these critically ill patients [41]. It is crucial to recognize that while refractory respiratory failure was also observed in the CRRT group, the elevated mortality rate in these patients might not be solely due to CRRT. Instead, it likely reflects the overall severity of their condition. This highlights the importance of CRRT in managing severe hemodynamic instability, but also underscores the necessity for further research to discern the full extent of CRRT’s impact on patient outcomes in severe COVID-19 cases.

Our study had several limitations. First, direct comparisons of the laboratory findings and vital signs immediately before CRRT initiation were unavailable. However, in most centers, CRRT initiation was determined through consultation with nephrologists, and we were able to compare the initial data, which helped to compensate for this limitation. Second, we did not collect data on the specific CRRT settings, including the mode and volume state, at each center, nor did we gather laboratory findings and vital signs before and after the initiation of CRRT. Therefore, our study lacks detailed information on the adjustment of CRRT settings at each center as well as crucial clinical data surrounding the initiation of therapy. Third, as a multicenter study, there may have been variations in patient populations across different centers. Nevertheless, by collecting data from multiple institutions, we assembled a dataset that is more representative of patients with COVID-19 on MV, allowing for a higher likelihood of capturing the characteristics of this patient group. Fourth, our study has the heterogeneity within the non-CRRT group. This group encompasses both patients without acute kidney injury and those with AKI who did not receive CRRT. This categorization may dilute specific findings and affect the clarity of our conclusions, suggesting a need for more distinct subgroup analysis in future research.

## Conclusions

In our study, 19.9% of COVID-19 patients requiring mechanical ventilation underwent CRRT. These patients demonstrated a higher mortality rate compared to those not receiving CRRT, with baseline CKD, higher BMI, and elevated WBC counts and BUN levels being factors necessitating CRRT. Although a higher mortality rate was observed in the CRRT group, primarily due to multi-organ failure and cardiac events, we found no significant difference in deaths due to respiratory failure between the CRRT and non-CRRT groups. This may underscore the multifactorial nature of mortality in these critically ill populations. These findings emphasize the importance of these factors in managing COVID-19 patients on mechanical ventilation and highlight the need for further research to better understand and address the risks associated with CRRT in this patient population.

## Supporting information

S1 Table Participating hospital name. (DOCX)

S2 Table Initial vital sign and findings of enrolled patients. (DOCX)

S3 Table Univariate and multivariate risk factors associated with in-hospital mortality (Cox regression analysis). (DOCX)

S4 Table Initial vital sign and findings of enrolled patients—non-survived patients. (DOCX)
