# Supplementary material for: Clinical characteristics and prognosis of patients with COVID-19 on mechanical ventilation undergoing continuous renal replacement therapy
Source: PLoS One. 2024 Apr 3;19(4):e0297344. doi: 10.1371/journal.pone.0297344 (PMC10990228; doi:10.1371/journal.pone.0297344)
Supplement: S1 Table — (DOCX) [file pone.0297344.s001.docx]

S1 Table. Participating hospital name

|  | Hospital | Name |
| --- | --- | --- |
| 1 | Pusan National University Yangsan Hospital | Woo Hyun Cho |
| 2 | Asan Medical Center | Huh Jin-Won |
| 3 | Chung-ang University hospital | Moon Seong Baek |
| 4 | Severance hospital | Su Hwan Lee |
| 5 | Seoul national university hospital | Sang-Min Lee |
| 6 | Samsung Medical Center | Chi Ryang Chung |
| 7 | The Catholic University, ST. Mary’s Hospital | Jongmin Lee |
| 9 | Inha University Hospital | Jung Soo Kim |
| 10 | Seoul National University Bundang Hospital | Sung Yoon Lim |
| 11 | Soonchunhyang University Bucheon Hospital | Ae-Rin Baek |
| 12 | Hallym University Medical Center | Sunghoon Park |
| 13 | Gyeongsang National University Hospital | Jung-Wan Yoo |
| 14 | Gyeongsang National University Changwon Hospital | Ho Cheol Kim |
| 15 | Yeungnam University Hospital | Eun Young Choi |
| 17 | Wonkang University Hospital | Chul Park |
| 19 | Chonnam National University Hospital | TaeOk Kim |
| 20 | Chosun University Hospital | Do Sik Moon |
| 21 | Chungnam National University Hospital | Song I Lee |
| 22 | Chungnam National University Sejong Hospital | Jae Young Moon |
| 23 | Konyang University Hospital | Sun Jung Kwon |
| 24 | Jeju National University Hospital | Gil Myeong Seong |
| 6 | Korea University Anam Hospital | Won Jai Jung |
