# Supplementary material for: Clinical characteristics and prognosis of patients with COVID-19 on mechanical ventilation undergoing continuous renal replacement therapy
Source: PLoS One. 2024 Apr 3;19(4):e0297344. doi: 10.1371/journal.pone.0297344 (PMC10990228; doi:10.1371/journal.pone.0297344)
Supplement: S2 Table — (DOCX) [file pone.0297344.s002.docx]

Table S2. Initial vital sign and findings of enrolled patients

| Variables | All patients  (n = 640) | Non-CRRT  (n = 517) | CRRT  (n = 123) | p-value |
| --- | --- | --- | --- | --- |
| Initial vital sign |  |  |  |  |
| Systolic BP, mmHg | 132.8 ± 24.6 | 132.9 ± 24.1 | 132.4 ± 27.0 | 0.860 |
| Diastolic BP, mmHg | 75.3 ± 15.0 | 75.6 ± 14.9 | 74.5 ± 15.4 | 0.478 |
| Heart rate, /min | 78.2 ± 26.5 | 77.8 ± 25.9 | 79.9 ± 29.0 | 0.424 |
| Respiratory rate, /min | 23.7 ± 6.3 | 23.7 ± 6.4 | 23.7 ± 5.7 | 0.980 |
| Body temperature (℃) | 37.0 ± 0.9 | 37.0 ± 0.8 | 36.9 ± 0.9 | 0.080 |
| Laboratory findings |  |  |  |  |
| White blood cell, 10^3^/uL | 9.6 ± 7.7 | 9.0 ± 5.3 | 12.2 ± 13.7 | 0.015 |
| Hemoglobin, g/dL | 12.9 ± 2.0 | 13.0 ± 1.9 | 12.6 ± 2.5 | 0.128 |
| Platelet, 10^3^/uL | 188.1 ± 78.2 | 189.8 ± 79.3 | 181.1 ± 73.1 | 0.266 |
| Albumin, g/dL | 3.2 ± 0.5 | 3.2 ± 0.5 | 3.1 ± 0.5 | 0.008 |
| Bilirubin, mg/dL | 0.71 ± 0.50 | 0.71 ± 0.51 | 0.68 ± 0.43 | 0.515 |
| BUN, mg/dL | 25.5 ± 17.9 | 22.0 ± 12.3 | 40.2 ± 27.8 | <0.001 |
| Creatinine, mg/dL | 1.24 ± 1.64 | 0.97 ± 1.02 | 2.39 ± 2.83 | <0.001 |
| PT, sec | 13.1 ± 3.4 | 13.0 ± 3.3 | 13.6 ± 4.0 | 0.073 |
| C-reactive protein, mg/dL | 10.1 (4.9 – 17.5) | 9.6 (4.8 – 16.9) | 12.5 (5.1 – 19.7) | 0.814 |
| Arterial blood gas analysis | | | | |
| pH | 7.41 ± 0.09 | 7.43 ± 0.08 | 7.36 ± 0.11 | <0.001 |
| PaCO2, mmHg | 35.1 ± 9.1 | 34.9 ± 8.4 | 36.0 ± 11.4 | 0.338 |
| PaO2, mmHg | 82.2 ± 34.0 | 81.9 ± 33.4 | 83.2 ± 36.7 | 0.720 |
| P/F ratio, mmHg | 149.6 ± 99.2 | 152.4 ± 100.6 | 137.8 ± 92.9 | 0.148 |
| Lactate, mmol/L | 1.69 (1.20 – 2.30) | 1.60 (1.16 – 2.20) | 2.05 (1.30 – 2.82) | 0.239 |

Data are presented as mean ± standard deviation or median and interquartile range, unless otherwise indicated.

BP: blood pressure, BUN: blood urea nitrogen, PT: prothrombin time, pH: potential of hydrogen, PaCO2: partial pressure of carbon dioxide in alveolar gas, PaO2: partial pressure of oxygen in arterial blood, P/F ratio: arterial partial pressure of oxygen/inspired oxygen concentration ratio
