# Supplementary material for: Clinical characteristics and prognosis of patients with COVID-19 on mechanical ventilation undergoing continuous renal replacement therapy
Source: PLoS One. 2024 Apr 3;19(4):e0297344. doi: 10.1371/journal.pone.0297344 (PMC10990228; doi:10.1371/journal.pone.0297344)
Supplement: S3 Table — (DOCX) [file pone.0297344.s003.docx]

Table S3. Univariate and multivariate risk factors associated with in-hospital mortality (Cox regression analysis)

|  | Univariate analysis | | | Multivariate analysis | | |
| --- | --- | --- | --- | --- | --- | --- |
|  | HR | 95% CI | P-value | HR | 95% CI | P-value |
| Age | 1.032 | 1.020 – 1.044 | <0.001 | 1.008 | 0.995 – 1.020 | 0.225 |
| Male | 1.029 | 0.796 – 1.329 | 0.828 |  |  |  |
| Body mass index | 1.010 | 0.980 – 1.040 | 0.513 |  |  |  |
| Clinical frailty scale | 1.080 | 1.004 – 1.161 | 0.038 | 0.965 | 0.873 – 1.068 | 0.491 |
| SOFA score | 1.066 | 1.026 – 1.108 | 0.001 | 0.996 | 0.951 – 1.042 | 0.848 |
| Comorbidity |  |  |  |  |  |  |
| Hypertension | 1.126 | 0.875 – 1.449 | 0.357 |  |  |  |
| Diabetes | 1.034 | 0.800 – 1.336 | 0.799 |  |  |  |
| Chronic lung disease | 1.309 | 0.877 – 1.953 | 0.187 |  |  |  |
| Chronic kidney disease | 1.580 | 1.090 – 2.291 | 0.016 | 1.119 | 0.656 – 1.907 | 0.680 |
| Solid tumor | 1.526 | 1.023 – 2.276 | 0.039 | 0.913 | 0.572 – 1.457 | 0.701 |
| Laboratory findings |  |  |  |  |  |  |
| White blood cell, 10^3^/uL | 1.004 | 0.993 – 1.016 | 0.466 |  |  |  |
| Hemoglobin, g/dL | 0.934 | 0.880 – 0.991 | 0.023 | 0.989 | 0.924 – 1.058 | 0.743 |
| Platelet, 10^3^/uL | 0.999 | 0.997 – 1.000 | 0.106 |  |  |  |
| Albumin, g/dL | 0.900 | 0.714 – 1.134 | 0.370 |  |  |  |
| Bilirubin, mg/dL | 1.248 | 1.023 – 1.522 | 0.029 | 1.391 | 1.124 – 1.723 | 0.002 |
| Creatinine, mg/dL | 1.117 | 1.059 – 1.179 | <0.001 | 1.118 | 1.037 – 1.207 | 0.004 |
| C-reactive protein, mg/dL | 1.001 | 1.000 – 1.002 | 0.169 |  |  |  |
| P/F ratio, mmHg | 0.999 | 0.998 – 1.001 | 0.227 |  |  |  |
| Lactate, mmol/L | 1.044 | 1.017 – 1.072 | 0.002 | 1.012 | 0.981 – 1.043 | 0.450 |
| Treatment |  |  |  |  |  |  |
| Steroid | 0.901 | 0.445 – 1.823 | 0.771 |  |  |  |
| Tocilizumab | 0.943 | 0.591 – 1.506 | 0.806 |  |  |  |
| CRRT | 2.789 | 2.164 – 3.595 | <0.001 | 2.228 | 1.648 – 3.014 | <0.001 |
| LST issue | 6.779 | 5.180 – 8.870 | <0.001 | 6.084 | 4.509 – 8.210 | <0.001 |

HR: Hazard ratio, CI: Confidence interval, SOFA: Sequential Organ Failure Assessment, P/F ratio: arterial partial pressure of oxygen/inspired oxygen concentration ratio, CRRT: continuous renal replacement therapy, LST: life sustaining treatment
