# Supplementary material for: Clinical characteristics and prognosis of patients with COVID-19 on mechanical ventilation undergoing continuous renal replacement therapy
Source: PLoS One. 2024 Apr 3;19(4):e0297344. doi: 10.1371/journal.pone.0297344 (PMC10990228; doi:10.1371/journal.pone.0297344)
Supplement: S4 Table — (DOCX) [file pone.0297344.s004.docx]

Table S4. Initial vital sign and findings of enrolled patients - non-survived patients

| Variables | All patients  (n = 251) | Non-CRRT  (n = 153) | CRRT  (n = 98) | p-value |
| --- | --- | --- | --- | --- |
| Initial vital sign |  |  |  |  |
| Systolic BP, mmHg | 132.0 ± 25.8 | 131.7 ± 25.5 | 132.3 ± 26.3 | 0.858 |
| Diastolic BP, mmHg | 73.9 ± 15.3 | 73.9 ± 15.3 | 74.0 ± 15.2 | 0.937 |
| Heart rate, /min | 77.4 ± 27.3 | 77.5 ± 25.8 | 77.3 ± 29.6 | 0.947 |
| Respiratory rate, /min | 23.4 ± 6.4 | 22.9 ± 6.5 | 24.1 ± 6.0 | 0.169 |
| Body temperature (℃) | 36.9 ± 0.9 | 36.8 ± 0.9 | 36.9 ± 0.9 | 0.524 |
| Laboratory findings |  |  |  |  |
| White blood cell, 10^3^/uL | 10.3 ± 10.1 | 9.2 ± 5.8 | 12.0 ± 14.3 | 0.034 |
| Hemoglobin, g/dL | 12.6 ± 2.0 | 12.6 ± 2.2 | 12.6 ± 1.8 | 0.823 |
| Platelet, 10^3^/uL | 176.7 ± 78.3 | 174.5 ± 81.7 | 180.0 ± 73.0 | 0.590 |
| Albumin, g/dL | 3.1 ± 0.5 | 3.2 ± 0.6 | 3.1 ± 0.5 | 0.947 |
| Bilirubin, mg/dL | 0.76 ± 0.55 | 0.80 ± 0.60 | 0.71 ± 0.44 | 0.395 |
| BUN, mg/dL | 30.8 ± 21.8 | 25.1 ± 14.7 | 39.5 ± 27.6 | 0.222 |
| Creatinine, mg/dL | 1.54 ± 2.06 | 1.11 ± 1.15 | 2.22 ± 2.85 | <0.001 |
| PT, sec | 13.4 ± 3.3 | 13.2 ± 2.7 | 13.7 ± 4.1 | 0.272 |
| C-reactive protein, mg/dL | 10.9 (5.6 – 18.8) | 10.0 (5.5 – 17.4) | 12.9 (5.6 – 20.1) | 0.540 |
| Arterial blood gas analysis | | | | |
| pH | 7.40 ± 0.10 | 7.42 ± 0.09 | 7.36 ± 0.11 | <0.001 |
| PaCO2, mmHg | 35.5 ± 10.8 | 35.3 ± 10.7 | 35.8 ± 10.9 | 0.716 |
| PaO2, mmHg | 78.7 ± 31.5 | 76.2 ± 30.2 | 82.7 ± 33.3 | 0.116 |
| P/F ratio, mmHg | 137.0 ± 83.3 | 140.5 ± 85.7 | 131.7 ± 79.6 | 0.419 |
| Lactate, mmol/L | 1.8 (1.2 – 2.7) | 1.6 (1.2 – 2.2) | 2.2 (1.4 – 2.9) | 0.608 |

Data are presented as mean ± standard deviation or median and interquartile range, unless otherwise indicated.

BP: blood pressure, BUN: blood urea nitrogen, PT: prothrombin time, pH: potential of hydrogen, PaCO2: partial pressure of carbon dioxide in alveolar gas, PaO2: partial pressure of oxygen in arterial blood, P/F ratio: arterial partial pressure of oxygen/inspired oxygen concentration ratio
